# Destabilization of vitelline membrane outer layer protein 1 homolog (VMO1) by *C*‐mannosylation

**DOI:** 10.1002/2211-5463.13561

**Published:** 2023-01-30

**Authors:** Satoshi Yoshimoto, Takehiro Suzuki, Naoki Otani, Daisuke Takahashi, Kazunobu Toshima, Naoshi Dohmae, Siro Simizu

**Affiliations:** ^1^ Department of Applied Chemistry, Faculty of Science and Technology Keio University Yokohama Japan; ^2^ Biomolecular Characterization Unit RIKEN Center for Sustainable Resource Science Wako Japan

**Keywords:** *C*‐mannosylation, mass spectrometry, protein stability, secretion, VMO1

## Abstract

*C*‐mannosylation is a rare type of protein glycosylation whereby a single mannose is added to the first tryptophan in the consensus sequence Trp‐Xaa‐Xaa‐Trp/Cys (in which Xaa represents any amino acid). Its consensus sequence is mainly found in proteins containing a thrombospondin type‐1 repeat (TSR1) domain and in type I cytokine receptors. In these proteins, *C*‐mannosylation affects protein secretion, intracellular localization, and protein stability; however, the role of *C*‐mannosylation in proteins that are not type I cytokine receptors and/or do not contain a TSR1 domain is less well explored. In this study, we focused on human vitelline membrane outer layer protein 1 homolog (VMO1). VMO1, which possesses two putative *C*‐mannosylation sites, is a 21‐kDa secreted protein that does not contain a TSR1 domain and is not a type I cytokine receptor. Mass spectrometry analyses revealed that VMO1 is *C*‐mannosylated at Trp^105^ but not at Trp^44^. Although *C*‐mannosylation does not affect the extracellular secretion of VMO1, it destabilizes the intracellular VMO1. In addition, a structural comparison between VMO1 and *C*‐mannosylated VMO1 showed that the modification of the mannose changes the conformation of three loops in VMO1. Taken together, our results demonstrate the first example of *C*‐mannosylation for protein destabilization of VMO1.

## Graphical abstract

Human VMO1 possesses two putative C‐mannosylation sites. Mass spectrometry analyses showed that VMO1 is *C*‐mannosylated at Trp^105^ but not at Trp^44^. When a single mannose is attached to Trp^105^ of VMO1, Maestro predicted that the structural conformation of VMO1 changes. In addition, *C*‐mannosylation destabilized VMO1. This study is the first example of *C*‐mannosylation leading to protein destabilization.

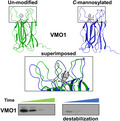

**Abbreviations**
CBB: Coomassie Brilliant Blue
CHX: Cycloheximide
CM: conditioned media
DMEM: Dulbecco's modified Eagle's medium
ER: endoplasmic reticulum
RMSD: root mean square deviation
TSR1: thrombospondin type‐1 repeat
VMO1: vitelline membrane outer layer protein 1

*C*‐mannosylation is a type of protein glycosylation that occurs at the indole‐C2 position of tryptophan in target proteins possessing the consensus motif in the endoplasmic reticulum (ER). Since *C*‐mannosylation was first detected in RNase2 from human urine, approximately 30 *C*‐mannosylation proteins have been reported [1, 2]. These proteins are classified into two broad categories: TSR1 superfamily and type I cytokine receptors. *C*‐mannosylation is important for the secretion, intracellular localization, and stability of proteins belonging to these two categories [3]. In general, *C*‐mannosylation assists intracellular trafficking from ER to the Golgi apparatus and secretion of substrate proteins [4, 5, 6, 7, 8, 9, 10]. *C*‐mannosyltryptophan residues, forming the Trp‐Arg ladder in the TSR1 domain, support the folding process and enhance the stability of the folded UNC‐5 protein, a TSR1 domain‐containing netrin receptor [11]. There are other examples of *C*‐mannosylation affecting protein structure. In RNase 2, the most abundant of the ^1^C_4_ conformation is exist among the C‐linked mannopyranosyl residue, and this conformation is predicted to stabilize protein folding through a network of hydrogen bonds [12]. Similarly, *C*‐mannosylation has been shown to enhance protein stability in RAMP1 [13]. Among reported *C*‐mannosylated proteins, UNC‐5 is the most well‐studied about the relationship between *C*‐mannosylation and protein stability.

In the hen's egg, vitelline membrane outer layer protein 1 (VMO1) was detected in the outer layer of the vitelline membrane [14]. It is a component of the outer layer membrane and binds strongly to ovomucin [15]. The 3D structure of chicken VMO1 was resolved by x‐ray crystallography and is a type of all β‐protein consisting of 6 long β‐strands and 8 short β‐strands [16, 17]. This protein has 59.1% homology to human VMO1 protein [18]. Concerning the biological function of chicken VMO1, it possesses antibacterial activity in chicken amniotic fluid [19]. Also, chicken VMO1 is abundant in ovarian carcinomas of laying hens and is regulated by estrogen and microRNAs in the chicken oviduct [20]. Thus, chicken VMO1 may be a potential biomarker of ovarian carcinomas. Human VMO1 interacts with lysozyme C and stabilizes tear films [21]. Also, it was observed in urine samples from patients with esophagogastric junctional cancers [22]. Interestingly, nothing has been reported that chicken VMO1 and human VMO1 are glycosylated, even though these VMO1 are secreted proteins. Human VMO1 possesses two putative *C*‐mannosylation sites, but it has not been elucidated whether human VMO1 is *C*‐mannosylated or not.

In this study, we demonstrated that human VMO1 overexpressed is *C*‐mannosylated at Trp^105^ but not at Trp^44^. This modification did not alter the extracellular secretion; however, it destabilizes the intracellular VMO1. Our results provide the first report that *C*‐mannosylation destabilizes the modified protein.

## Material and methods

### Cell culture

The HT1080 (JCRB Cell Bank, Osaka, Japan) and HEK293T cells (RIKEN BioResource Center, Tsukuba, Japan) were cultured in Dulbecco's modified Eagle's medium (DMEM) (Nissui, Tokyo, Japan), supplemented with 10% (v/v) FBS, 100 units·mL^−1^ penicillin G, 100 mg·L^−1^ kanamycin, 600 mg·L^−1^ l‐glutamine, and 2.25 g·L^−1^ NaHCO_3_, under 5% CO_2_ in a humidified incubator at 37 °C [23].

### Plasmid construction

The synthetic DNA encoding C‐terminally myc‐his_6_‐tagged human VMO1 was purchased from GENE WIZ Japan Corp (Shinagawa‐ku, Tokyo, Japan) and was subcloned into CSII‐CMV‐MCS‐IRES2‐Bsd (RIKEN BioResource Center). The sequences of the tags were as follows: myc: 5′‐GAACAAAAACTCATCTCAGAAGAGGATCTG‐3′ and his_6_: 5′‐CATCATCACCATCACCAT‐3′. We amplified VMO1 using the following primers: 5′‐TTTTAGATCTATGGAGCGGGGCGCAGGAGC‐3′ (forward) and 5′‐TTTTGTCGACACTGCGGCAGCAGAATAAGC‐3′ (reverse) for full‐length VMO1. To obtain the VMO1‐GFP‐his_8_ expression vector, we introduced the His_8_‐tag into the pAcGFP1‐N1 vector (Addgene, Watertown, MA). The sequence of the his_8_ tag was as follows: his_8_: 5′‐CACCACCACCATCATCATCATCAT‐3′. The obtained VMO1‐GFP‐his_8_ cDNA was subcloned into CSII‐CMV‐MCS‐IRES2‐Bsd vector. The sequences of the primers that were used for the mutagenesis were as follows: 5′‐GTCTGGAAGCTTCGGCGAATGGAG‐3′ (forward) and 5′‐CTCCATTCGCCGAAGCTTCCAGAC‐3′ (reverse).

### Establishment of VMO1‐overexpressing cell line and purification of recombinant VMO1

To establish VMO1‐overexpressing HT1080 cell line, the CSII‐CMV‐MCS‐IRES2‐Bsd‐VMO1 or CSII‐CMVMCS‐IRES2‐Bsd‐luciferase plasmid was transfected into HEK293T cells using the Lentivirus High Titer Packaging Mix (Takara Bio Inc., Shiga, Japan) for lentivirus production. After 6 h of transfection, the cells were washed with PBS and cultured in serum‐free DMEM for 48 h. After 48 h, the conditioned medium containing lentivirus was collected, and HT1080 cells were infected with the collected medium and cultured for 24 h. After infection, the cells were washed with PBS and were selected with 10 μg·mL^−1^ blasticidin S (FUJIFILM Wako Pure Chemical Corp.). The clones that expressed high levels of wild‐type VMO1‐GFP‐his_8_, wild‐type VMO1‐myc‐his_6_, and mutant VMO1 (W105F)‐myc‐his_6_ were designated HT1080‐VMO1‐GH, HT‐VMO1‐MH, and HT1080‐VMO1/W105F‐MH cells, respectively. HT1080 cells transfected with GFP vector were termed HT1080‐GFP.

To purify the recombinant VMO1 protein, HT1080‐VMO1‐GH cells were cultured in serum‐free DMEM for 24 h. The conditioned medium was collected and concentrated using Ultra 15 mL filters (Merck KGaA, Darmstadt, Germany). The concentrated sample was incubated with Ni‐NTA agarose beads for 2 h at 4 °C. Ni‐NTA‐bound proteins were eluted with 100 mm imidazole, and the eluates were electrophoresed on an SDS/polyacrylamide gel. The protein bands were visualized with Coomassie Brilliant Blue (CBB) R‐250 (FUJIFILM Wako Pure Chemical Corporation, Ltd., Osaka, Japan).

### Mass spectrometry

Purified VMO1 was analyzed by SDS/PAGE. After CBB staining, the visible band was excised and destained. The gel pieces were treated with DTT and acrylamide to reduce and alkylate, followed by trypsin digestion (TPCK treated; Worthington Biochemical, Worthington, OH, USA).

The resulting peptides were analyzed using MALDI‐TOF MS (rapifleX MALDI Tissuetyper, Bruker Daltonics, Bremen, Germany). MALDI‐TOF MS was performed in positive reflector mode using α‐cyano‐4‐hydroxycinnamic acid as a matrix. Subsequently, the peptide mixture was subjected to LC–MS/MS (Q Exactive, Thermo Fisher Scientific, Inc., Waltham, MA, USA) with the data‐dependent Top 10 method. Solvent A (0.1% formic acid) and solvent B (100% acetonitrile with 0.1% formic acid) were used as eluents. Peptides were separated using an Easy nLC 1000 (Thermo Fisher Scientific, Inc., Waltham, MA, USA) equipped with a nano‐ESI spray column (NTCC‐360, 0.075 mm internal diameter × 105 mm length, 3 μm, Nikkyo Technos Co, Bunkyo‐ku, Tokyo, Japan) at a flow rate of 300 nL·min^−1^ under a linear gradient condition over 20 min. The acquired data were processed using MASCOT 2.7 (Matrix Science, London, UK) and Proteome Discoverer 2.4 (Thermo Fisher Scientific, Inc., Waltham, MA, USA). The MASCOT parameters were as follows: Database, in‐house database including VMO1 sequence; type of search, MS/MS ion; enzyme, trypsin; fixed modification, none; variable modifications, Hex (W), acetyl (protein N‐term), Gln‐ > pyro‐Glu (N‐term Q), oxidation (M), propionamide (C) deamidation (NQ); mass values, monoisotopic; peptide mass tolerance, ± 15 ppm; fragment mass tolerance, ± 30 mmu; max missed cleavages, 3; and instrument type, ESI‐TRAP. The MS chromatograms and MS/MS spectra were drawn using Qual Browser software (Xcalibur®, version 4.1.50, Thermo Fisher Scientific, Inc., Waltham, MA, USA).

### Western blot

To detect the VMO1 protein in cell lysates and conditioned media (CM), cells were cultured in serum‐free medium for 24 h and lysed in lysis buffer [50 mm Tris–HCl (pH 7.5), 150 mm NaCl, 0.1% (w/v) SDS, 1% (v/v) Triton X‐100, 1% (w/v) sodium deoxycholate, and 1 mm phenylmethylsulfonyl fluoride] at 4 °C with sonication. The lysates were centrifuged at 15,300 ***g*** for 10 min and equalized for protein concentration for each cell lysate. The samples were boiled at 98 °C in loading buffer [350 mm Tris–HCl (pH 6.8), 30% (w/v) glycerol, 0.012% (w/v) bromophenol blue, 6% (w/v) SDS, and 30% (v/v) 2‐mercaptoethanol] for 5 min and electrophoresed on SDS/polyacrylamide gels. Proteins were transferred to a polyvinylidene fluoride membrane and sequentially immunoblotted with primary antibodies against c‐myc (mouse monoclonal, DSHB Hybridoma Product 9E10), GFP (mouse monoclonal, sc‐9996, Santa Cruz Biotechnology, Dallas, TX), and α‐tubulin (mouse monoclonal, #T5168, Merck KGaA). Horseradish peroxidase‐conjugated sheep polyclonal anti‐mouse IgG (GE Healthcare Life Sciences, Chicago, IL) was used as a secondary antibody. Signals were detected using Immobilon Western Chemiluminescent HRP substrate (Merck KGaA) [24, 25]. Protein bands were quantified in ImageJ (National Institutes of Health, Bethesda, MD).

### Semiquantitative RT‐PCR

Total RNA was extracted from cultured cells using TRIzol (Thermo Fisher Scientific, Inc., Waltham, MA), according to the manufacturer's instructions, and 2 μg of total RNA was used for the reverse‐transcription reaction with the High‐Capacity cDNA reverse‐transcription kit (Thermo Fisher Scientific, Inc.). The resulting cDNA was used for PCR amplification [26, 27]. The sequences of the primers, the number of cycles, and the annealing temperatures were as follows: *VMO1*: 5′‐TTTTGAATTCATGGAGCGGGGCGCAGGAGC‐3′ (forward) and 5′‐CTCCAGTCTCCAAAGTCTCC‐3′ (reverse), 25 cycles, and 63 °C; *GAPDH*: 5′‐GATTCCACCCATGGCAAATTCC‐3′ (forward) and 5′‐ CACGTTGGCAGTGGGGAC‐3′ (reverse), 20 cycles, and 63 °C.

### Cycloheximide chase assays

Around 80%‐confluent cells in 60 mm dishes were treated with 50 μg·mL^−1^ cycloheximide (CHX) (Millipore, Darmstadt, Germany) and incubated at several time points (0, 0.5, 1.0, and 1.5 h). After incubation, cells were lysed and analyzed by western blotting. The band intensities of wild‐type VMO1, VMO1 W105F, and α‐tubulin were quantified in imagej.

### Structural comparison

The PDB structure of VMO1 predicted by AlphaFold [28] was downloaded via UniProt. The structure was subjected to preparation steps using the Protein Preparation Wizard in Maestro, version 12.4 (Schrödinger, LLC 2020, New York, NY, USA) using the default settings. First, the waters beyond 5 Å from het groups were removed, bond orders were assigned, and hydrogens were added. Next, the optimization of the hydrogen bonding network was performed by reorienting hydroxyl and thiol groups, amide groups of Asn and Gln, and the imidazole ring in His and by predicting protonation states of His, Asp, and Glu and tautomeric states of His. In the final step, the energy minimization of the structure was carried out using Impref module of the Schrödinger suite with a cut of root mean square deviation (RMSD) of 0.30 Å. OPLS3e force field was used in the energy minimization phase. Then, amino acid residues 1–24 were deleted (Fig. 4A). Next, mannose was added to Trp^105^ in Fig. 4A, and minimization was performed using MacroModel, obtaining the intermediate structure (calculation conditions: force field: OPLS3e, solvent: water, freely moving atoms: all residues within 5 Å of the mannose, fixed atoms that are allowed to move a finite distance depending on the default force constant value: a shell of width 5 Å around the freely moving region, and frozen atoms: a shell of width 10 Å around the fixed region). Then, a loop refinement calculation was performed for loop 6 (amino acid residues 99–106) of the intermediate structure, obtaining Fig. 4B (calculation conditions: force field: OPLS3e, solvation model: VSGB). RMSD was calculated by superimposing the protein backbones of Fig. 4A,B (Fig. 4C,D). VMO1/W105F structure was prepared in the same way as wild‐type VMO1 structure.

## Results

### Human VMO1 is *C*‐mannosylated at Trp^105^ but not at Trp^44^

**Fig. 1:**
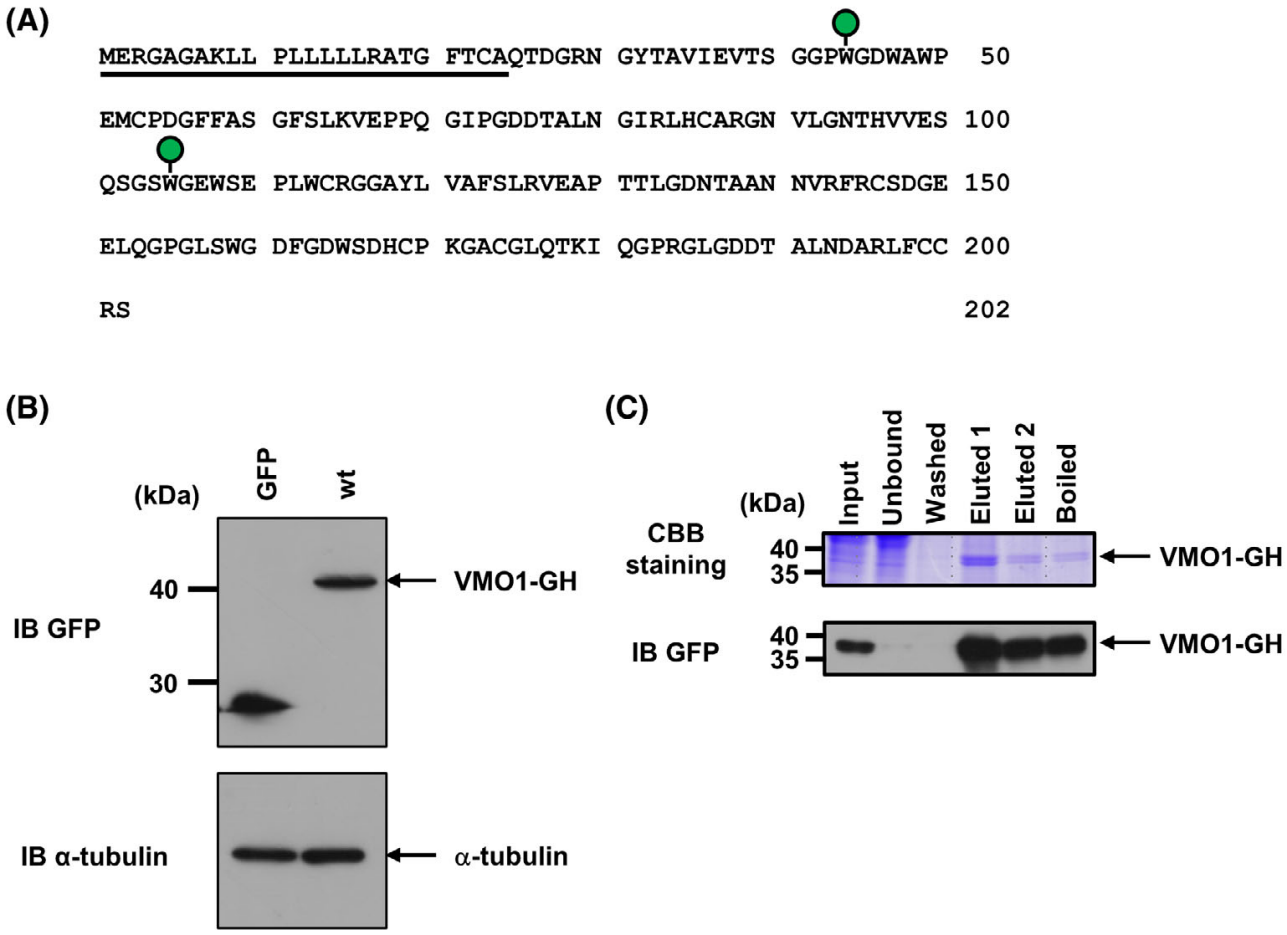
Establishment of VMO1‐overexpressing cell line for purification of recombinant VMO1. (A) Amino acid sequence of human VMO1. Human VMO1 is a 202‐a.a. protein. Underlined amino acid sequences indicate the signal peptide. Green circles indicate putative *C*‐mannosylation sites (Trp^44^ and Trp^105^). (B) Establishment of a VMO1‐overexpressing HT1080 cell line. HT1080‐GFP (GFP) and HT1080‐VMO1‐GH (wt) cells were cultured and lysed. Each total cell lysate was electrophoresed and immunoblotted with anti‐GFP and anti‐α‐tubulin. (C) Purification of recombinant human VMO1 from CMof HT1080‐VMO1‐GH cell line. Cells were cultured in serum‐free medium for 24 h, and CM was collected and concentrated using Ultra 15 mL filters. VMO1‐GH was purified with Ni‐NTA agarose beads. The samples were eluted, electrophoresed, and visualized by CBB staining in the gel.

**Fig. 2:**
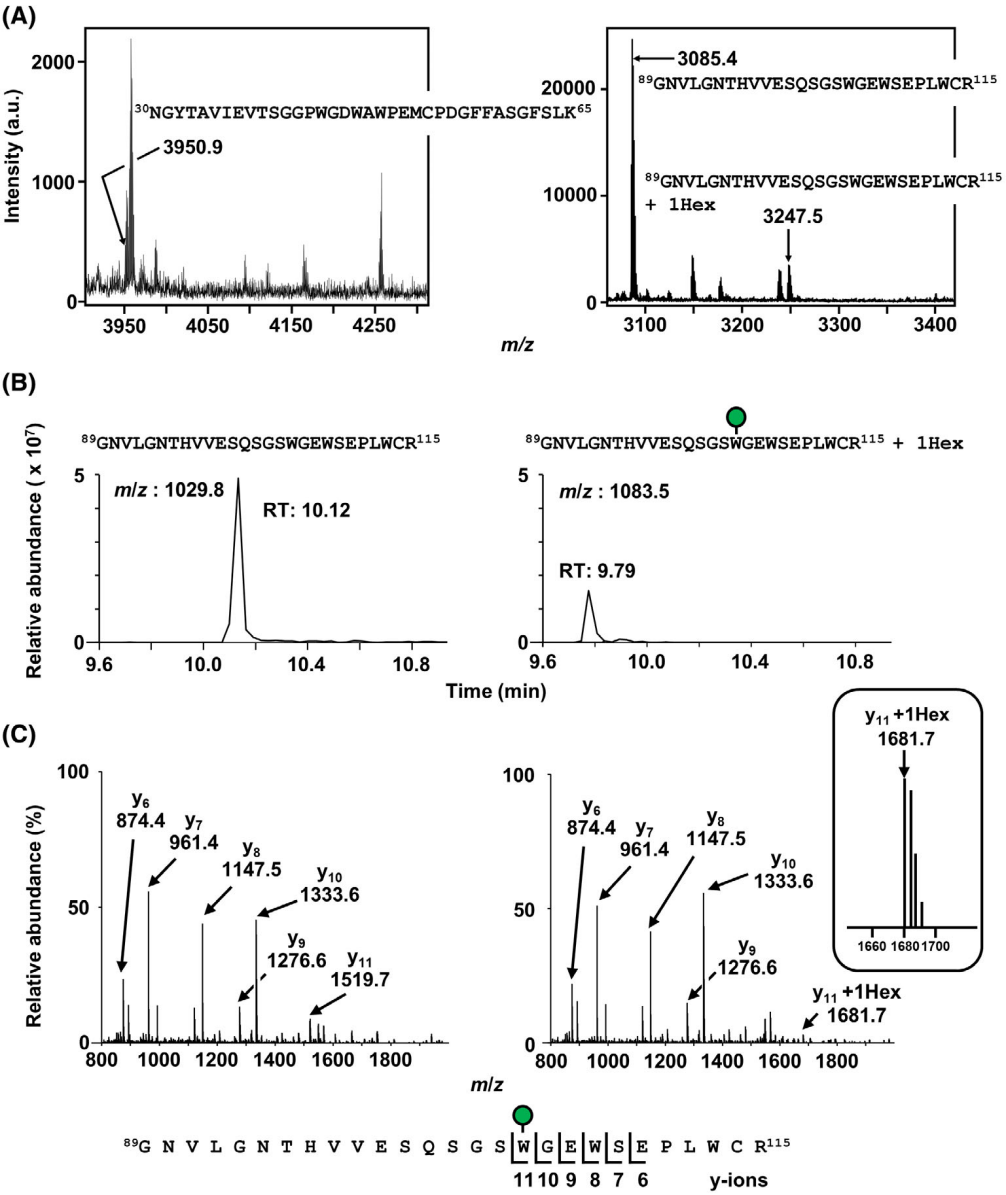
*C*‐mannosylation of VMO1 at Trp^105^. (A and B) Detection of *C*‐mannosylation peptide in VMO1. In‐gel digestion was performed using trypsin, and the resulting peptides were analyzed by MALDI‐TOF MS (A) and LC–MS (B). (C) The unmannosylated (left) and *C*‐mannosylated (right) ^89^GNVLGNTHVVESQSGSWGEWSEPLWCR^115^ peptides were detected in MS/MS spectra with parent ions of *m*/*z* 1029.8 and 1083.5, respectively. The indicated y ions were detected.

Human VMO1 contains two putative *C*‐mannosylation sites at Trp^44^ and Trp^105^ (Fig. 1A). To determine whether VMO1 is *C*‐mannosylated or not, we established a stable HT1080 cell line overexpressing C‐terminally GFP‐his8‐tagged VMO1, termed as HT1080‐VMO1‐GH (Fig. 1B). Using Ni‐NTA agarose beads, we purified recombinant VMO1 from the conditioned medium of the cell line to detect *C*‐mannosylation by MALDI‐TOF MS and LC–MS/MS (Fig. 1C). The purified sample was digested with trypsin and subsequently analyzed. As a result, the ^30^NGYTAVIEVTSGGPWGDWAWPEMCPDGFFASGFSLK^65^ peptide did not contain a hexose (*m*/*z* = 3950.9), suggesting that Trp^44^ residue is not *C*‐mannosylated (Fig. 2A upper). On the other hand, concerning the ^89^GNVLGNTHVVESQSGSWGEWSEPLWCR^115^ peptide, two forms, unmannosylated (*m*/*z* = 1029.8, R.T. = 10.12 min) and mono‐*C*‐mannosylated (*m*/*z* = 1083.5, R.T. = 9.79 min), were detected (Fig. 2A lower, 2B). To identify the *C*‐mannosylated site, these peptides were analyzed by MS/MS analysis. The *m*/*z* value of y_11_ ion from mannosylated peptide was increased by 162 (+1 hexose) compared with that from the unmannosylated peptide, while the *m*/*z* values of y_10_ ion from both unmannosylated and mono‐*C*‐mannosylated peptides were same (Fig. 2C). These results demonstrated that VMO1 is *C*‐mannosylated at Trp^105^ but not at Trp^44^.

### *C*‐mannosylation of VMO1 affects its stability but not secretion

**Fig. 3:**
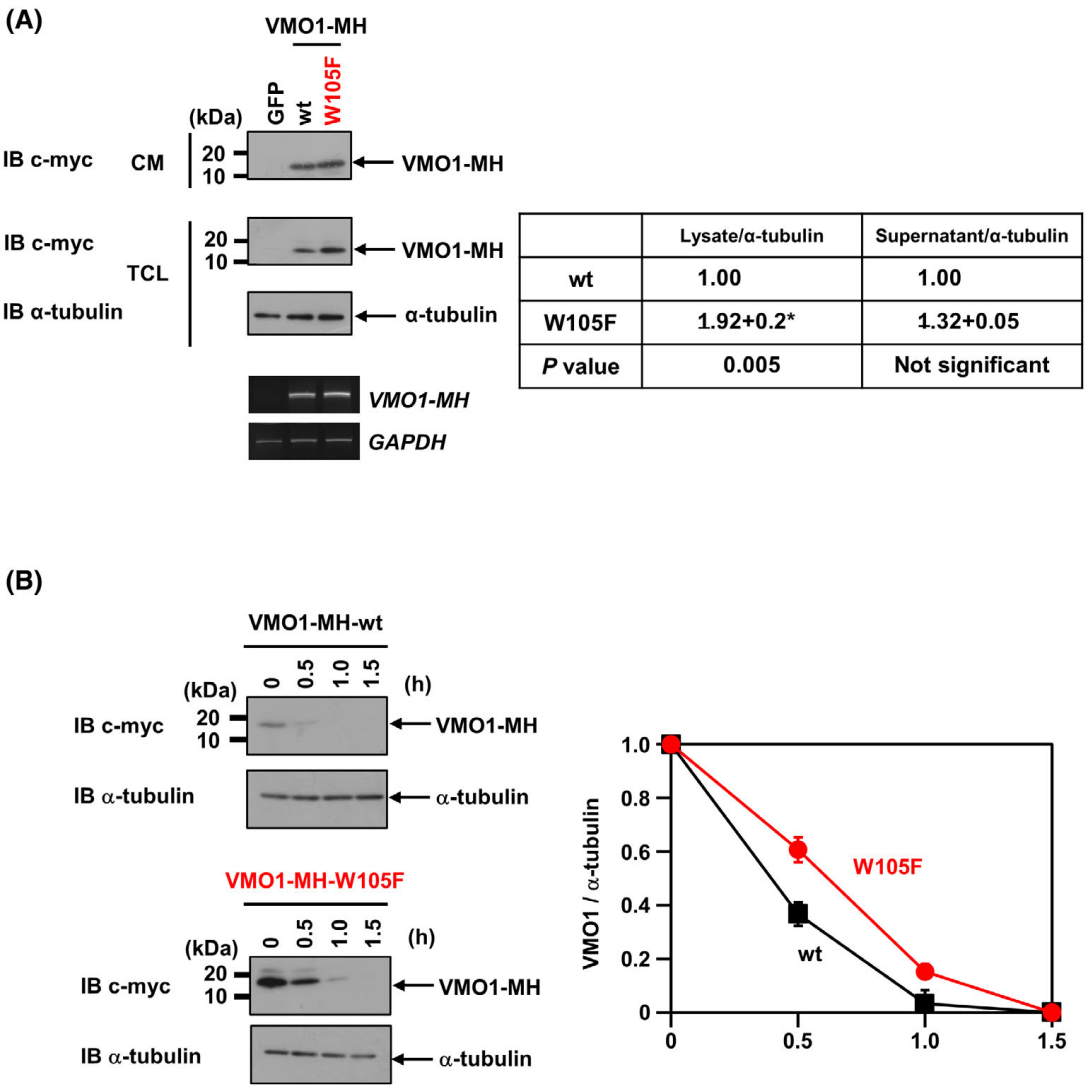
*C*‐mannosylation destabilizes the intracellular VMO1. (A) Establishment of *C*‐mannosylation‐defective VMO1 overexpression cell, HT1080‐VMO1/W105F‐MH. HT1080‐GFP (GFP), HT1080‐VMO1‐MH (wt), and HT1080‐VMO1/W105F‐MH (W105F) cells were cultured for a serum‐free medium for 24 h. Total cell lysates (TCLs) and conditioned media (CM) were collected. Equal amounts of proteins were electrophoresed and immunoblotted with anti‐c‐myc and anti‐α‐tubulin (top). Total RNAs were isolated from each cell line, and semiquantitative RT‐PCR was performed (bottom). Signal intensities (right) of the expression levels were quantified and normalized to α‐tubulin. The levels of each expression in HT1080‐VMO1‐MH cells were defined as 1.00 (mean + SD; *n* = 3). The “*” means a significant difference (*P* < 0.05). (B) The protein stability of VMO1 was analyzed by the CHX chase assay. HT1080‐VMO1‐MH (wt) and HT1080‐VMO1/W105F‐MH (W105F) cells were treated with CHX (50 μg·mL^−1^) for the indicated times. The cells were collected and lysed. Equal amounts of proteins were electrophoresed and immunoblotted with anti‐c‐myc and anti‐α‐tubulin (left). Signal intensities of the expression levels were quantified and normalized to α‐tubulin. The expression levels of each time point were plotted relative to the expression level at 0 h (mean + SD; *n* = 3) and graphed (right). The expression level at 0.5 h demonstrated a significant difference (*P* < 0.05).

*C*‐mannosylation also affects the secretion of its substrate proteins even if not belonging to proteins containing TSR1 domain and type I cytokine receptors [6, 8, 29]. To assess the effect of *C*‐mannosylation on the secretion of VMO1, we established stable HT1080 cell lines overexpressing C‐terminally Myc‐his_6_‐tagged wild‐type form of VMO1 and a *C*‐mannosylation‐defective mutant form of VMO1, termed as HT1080‐VMO1‐MH and HT1080‐VMO1/W105F‐MH, respectively (Fig. 3A). The secretion levels of VMO1 did not differ between wild‐type VMO1 and VMO1/W105F (Fig. 3A). However, the intracellular levels of VMO1/W105F were higher than that of wild‐type VMO1 (Fig. 3A). Since the mRNA levels of wild‐type VMO1 and VMO1/W105F were equivalent, it is suggested that the destabilization of *C*‐mannosylated VMO1 caused its difference.

To test whether *C*‐mannosylation destabilizes the intracellular VMO1, we performed the CHX chase assay. As shown in Fig. 3B, the wild‐type VMO1 protein level significantly decreased after 0.5 h of treatment, while VMO1/W105F protein level slightly decreased. Taken together, these results suggested that *C*‐mannosylation is critical for the stability of VMO1.

### *C*‐mannosylation is predicted to possibly impact the conformation of VMO1

**Fig. 4:**
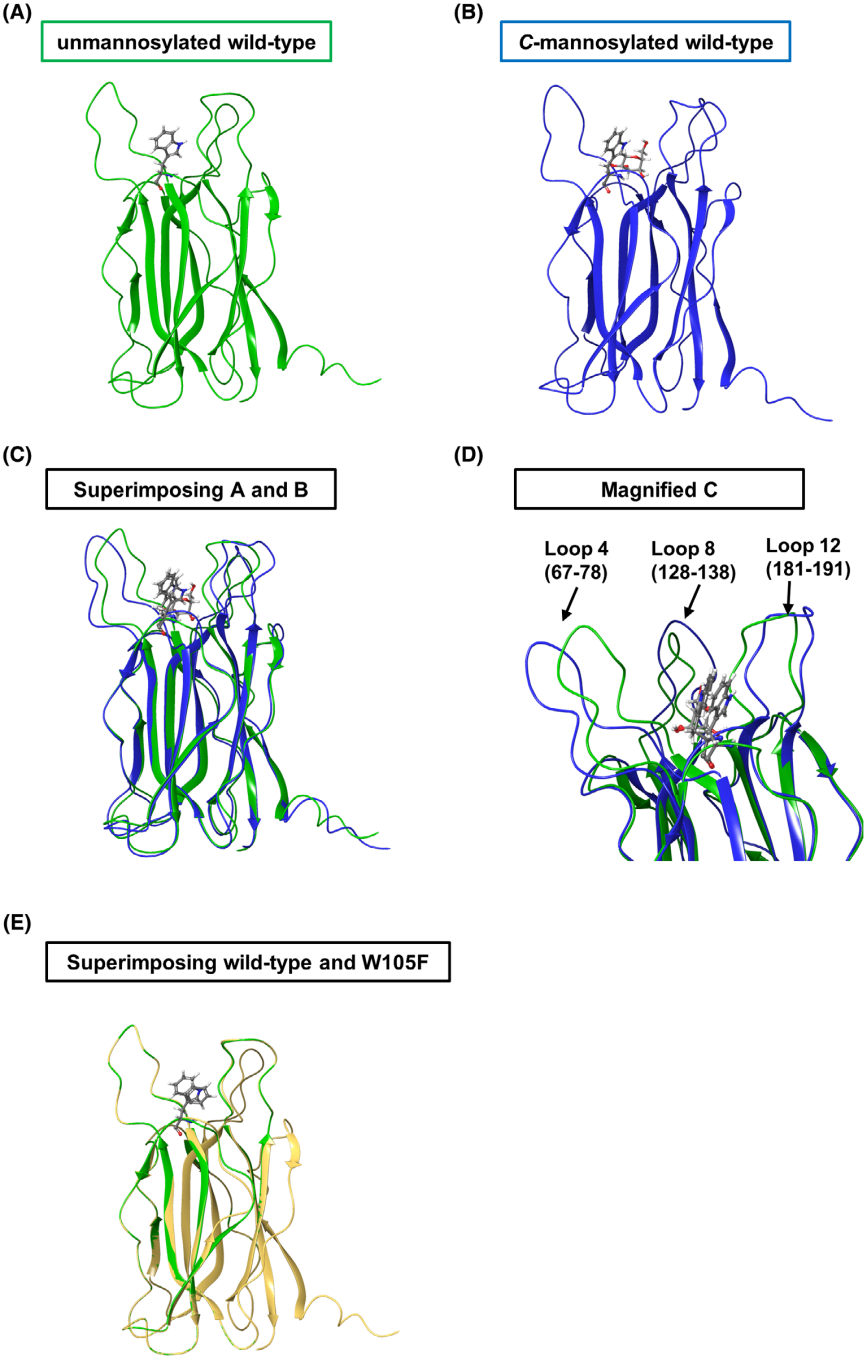
Structural comparison of unmannosylated wild‐type VMO1 and *C*‐mannosylated wild‐type VMO1. (A) Wild‐type VMO1 structure was in green. (B) Wild‐type VMO1 structure in complex with *C*‐mannosyltryptophan was in blue. (C and D) Superimposing unmannosylated structure and *C*‐mannosylated structure (C). Three loop conformations were enlarged (D). (E) Superimposing unmannosylated structure and VMO1/W105F structure.

Several reports have suggested that *C*‐mannosylation changes protein conformation [11, 12, 30, 31]. Since *C*‐mannosylation destabilized the intracellular VMO1 (Fig. 3B), we compared wild‐type VMO1 structures between unmannosylated and *C*‐mannosylated using Maestro, version 12.4 (Schrödinger, LLC 2020). Maestro can predict the conformational change of a peptide when a glycosylation or amino acid substitution is added to the peptide. Specifically, it is possible to predict the loss of binding affinity caused by structural changes due to amino acid substitutions [32], and to evaluate *O*‐GlcNAc hydrolase (OGA)‐peptide interactions by docking Maestro‐generated glycopeptides into the bacterial OGA structure [33]. The 3D structure of human VMO1 has not been reported; thus, the PDB structure of VMO1 predicted by AlphaFold [26] was downloaded via UniProt and used in this analysis. From the structural comparisons, the value of RMSD of superimposing unmannosylated and *C*‐mannosylated was calculated as 1.440 (Fig. 4A–D). It is noteworthy that the modification of the mannose was predicted to change the structural conformation of three loops (loop 4, 67–78 a.a.; loop 8, 128–138 a.a.; and loop 12, 181–191 a.a.) in VMO1 (Fig. 4D). The conformational changes modified by *C*‐mannosylation were presumed to cause the destabilization of the intracellular VMO1.

## Discussion

Over 30 *C*‐mannosylated proteins have been reported since 1994, but the biological function of *C*‐mannosylation is still covered in a veil. Since numerous substrate proteins belonging to the TSR1 superfamily were found, the functional analyses of *C*‐mannosylation related to TSR1 domain were deeply studied [2, 3]. Meanwhile, the role of *C*‐mannosylation in proteins not containing TSR1 domain has not been elucidated yet. For example, although *C*‐mannosylation negatively regulates the secretion of hyaluronidase 1 [31], *C*‐mannosylation positively regulates protein secretion in general [3]. Thus, it is suggested that *C*‐mannosylation occasionally shows the opposite effect on proteins.

We showed that *C*‐mannosylation destabilized the intracellular VMO1 (Fig. 3). In some *C*‐mannosylated proteins, *C*‐mannosylation enhances protein stability [11, 12, 13]. CHX chase assay revealed that *C*‐mannosylated RAMP1 is more stabilized than non‐*C*‐mannosylated RAMP1 [13]. Several reports have suggested that *C*‐mannosylation affects protein conformations. For example, *C*‐mannosyltryptophan of UNC‐5 is involved in intramolecular hydrogen bonding and limited the flexibility of the tryptophan‐arginine ladder in TSR1 domain, resulting that it showed high thermal stability [11]. *C*‐mannosylation of mindin modulated disulfide bond formation in the intracellular mindin to promote its secretion [34]. In RNase 2, ^1^C_4_ conformation was the most abundant when mannose is attached to the Trp residue of RNase 2, and this conformation optimally stabilized protein folding via hydrogen bonds [12]. But, it has not been reported that *C*‐mannosylation destabilized proteins, as in our study. We have compared conformations of VMO1 between the unmannosylated and the *C*‐mannosylated forms (Fig. 4). The formations of loop 4, loop 8, and loop 12 were predicted to be significantly changed by the *C*‐mannosylation (Fig. 4D). Compared conformations both wild‐type VMO1 and VMO1/W105F, RMSD was calculated as 0.171 (Fig. 4E), suggesting amino acid substitution from Trp to Phe did not affect the stability of VMO1. We speculated that proper conformation of these three loops is important for the stabilization of VMO1.

In conclusion, we demonstrated that VMO1 is *C*‐mannosylated at Trp^105^ and destabilized by the *C*‐mannosylation. This study provides new insights into the regulation of VMO1 secretion and contributes to a better understanding of *C*‐mannosylation and protein stability.

## Conflict of interest

The authors declare no conflict of interest.

## Author contributions

SY and SS designed the research; SY performed the cell‐based functional analysis; TS and ND performed MS analysis; NO, DT and KT performed molecular simulation. SY and SS wrote the manuscript, and all authors contributed to the discussion of the results.

## Data Availability

The data that support the findings of this study are available from the corresponding author (simizu@applc.keio.ac.jp) upon reasonable request.
